# Noncontact atomic force microscopy

**DOI:** 10.3762/bjnano.3.17

**Published:** 2012-02-29

**Authors:** Udo D Schwarz

**Affiliations:** 1 Department of Mechanical Engineering and Materials Science, Yale University, P.O. Box 208284, New Haven, CT 06520-8284, USA

No other method has opened the door to progress in nanoscience and nanotechnology as much as the introduction of scanning probe methods did in the 1980s, since they offer a way to *visualize* the nanoworld. For maximum impact, however, the ability to image and manipulate individual atoms is the key. Initially, scanning tunneling microscopy was the only scanning-probe-based method that was able to achieve this resolution. Atomic force microscopy (AFM), on the other hand, was quickly developed into a versatile tool with applications ranging from materials characterization in ultrahigh vacuum and nanofabrication under ambient conditions, to biological studies in liquids, but its resolution was limited to the nanometer scale.

The reason for this restriction resulted from the fact that the resolution in probe microscopy scales with the sharpness of the tip. In conventional AFM operational modes, a tip that is located at the end of a leaf spring (the so-called cantilever) is either dragged over the surface in permanent contact or gently taps the surface while vibrating, and, whichever mode is used, tips quickly blunt through either permanent or intermittent contact. Maintaining the atomic sharpness of an initially atomically sharp tip requires that the tip never touches the surface. But how can the tip know that the surface is there if it is not allowed to touch? This problem was solved in the 1990s through the realization that the attractive forces acting on the tip when it is in close proximity to the sample affect the resonance frequency of the cantilever even though it is not in actual contact with the surface. Noncontact atomic force microscopy (NC-AFM) makes use of this effect by tracking the shift of the cantilever resonance frequency due to the force field of the surface without ever establishing physical contact between the tip and sample. Much to the astonishment of many, changes induced by individual atoms turned out to induce frequency shifts that are large enough to be detected, and thus atomic-scale imaging with AFM became a reality.

Since the beginnings, almost two decades ago, NC-AFM has evolved into a powerful method that is able not just to image surfaces, but also to quantify tip–sample forces and interaction potentials as well as to manipulate individual atoms on conductors, semiconductors, and insulators alike. For the community to keep track of the rapid development in the field, a series of annual international conferences, starting in Osaka, Japan in 1998, has been established. The most recent conference from this series was held in Lindau, Germany, from September 18–22, 2011. Once again, substantial progress was presented; NC-AFM is now able to quantitatively map three-dimensional force fields of surfaces with atomic resolution in ultrahigh vacuum as well as in liquids, and methodological developments add more information to the measurements, for example, through the driving of higher cantilever harmonics or the recording of tunneling currents. For this Thematic Series of the *Beilstein Journal of Nanotechnology*, many of the presenters from the Lindau conference agreed to submit contributions in order to assemble a series that showcases the present state of the art in the field. I would like to thank all authors who have contributed their excellent original work to this series, all referees whose promptly provided reports have provided valuable suggestions for further improvements while keeping the publication times short, and the entire NC-AFM community for supporting the open access policy of the *Beilstein Journal of Nanotechnology*.

Udo D. Schwarz

New Haven, February 2012

